# Exploiting real-world data to monitor physical activity in patients with osteoarthritis: the opportunity of digital epidemiology^☆^

**DOI:** 10.1016/j.heliyon.2022.e08991

**Published:** 2022-02-22

**Authors:** Silvia Ravalli, Federico Roggio, Giovanni Lauretta, Michelino Di Rosa, Agata Grazia D'Amico, Velia D'agata, Grazia Maugeri, Giuseppe Musumeci

**Affiliations:** a Department of Biomedical and Biotechnological Sciences, Human Anatomy and Histology Section, School of Medicine, University of Catania, Via S. Sofia 87, 95123 Catania, Italy; b Department of Psychology, Educational Science and Human Movement, University of Palermo, Via Giovanni Pascoli 6, 90144 Palermo, Italy; c Department of Drug and Health Sciences, University of Catania, 95125 Catania, Italy; d Research Center on Motor Activities (CRAM), University of Catania, 95123 Catania, Italy; e Department of Biology, College of Science and Technology, Temple University, Philadelphia, PA 19122, USA

**Keywords:** Physical activity, Osteoarthritis, Digital epidemiology, Digital devices, Exercise

## Abstract

Osteoarthritis is a degenerative joint disease that affects millions of people worldwide. Current guidelines emphasize the importance of regular physical activity as a preventive measure against disease progression and as a valuable strategy for pain and functionality management. Despite this, most patients with osteoarthritis are inactive. Modern technological advances have led to the implementation of digital devices, such as wearables and smartphones, showing new opportunities for healthcare professionals and researchers to monitor physical activity and therefore engage patients in daily exercising. Additionally, digital devices have emerged as a promising tool for improving frequent health data collection, disease monitoring, and supporting public health surveillance. The leveraging of digital data has laid the foundation for developing a new concept of epidemiological study, known as "Digital Epidemiology". Analyzing real-world data can change the way we observe human behavior and suggest health interventions, as in the case of physical exercise and osteoarthritic patients. Furthermore, large-scale data could contribute to personalized and precision medicine in the future. Herein, an overview of recent clinical applications of wearables for monitoring physical activity in patients with osteoarthritis and the benefits of exploiting real-world data in the context of digital epidemiology are discussed.

## 1 Introduction

Evidence-based data tie physical inactivity and sedentary habits to non-communicable diseases, including diabetes, cardiovascular disorders, and obesity [1, 2]. Exercising is widely suggested as a valuable preventive strategy to avoid the onset and slow down the progression of several pathologies. The American College of Sports Medicine (ACSM) and the World Health Organization (WHO) recommend exercising at least 30–45 min every day to a total of 150–300 min per week [3, 4, 5]. Osteoarthritis (OA), is a degenerative disease of the articular cartilage that mainly affects older people, causing disability worldwide [6]. Current treatments include the use of non-steroidal anti-inflammatory drugs, opioid and non-opioid analgesics, intra-articular injections of steroids and hyaluronic acid, and surgical procedures [7]. However, side effects and contraindications of these treatments suggest considering adopting new non-pharmacological, regenerative, and behavioral approaches [8, 9]. Physical activity (PA) represents a low-cost and feasible strategy to preserve joint function, flexibility, decrease pain and fatigue, and improve balance and muscle strength [10]. Patients with OA are strongly recommended to be physically active, avoiding excessive load or strenuous training [8, 9]. Especially in the case of knee OA, the amount of activity performed by the lower limb influences the muscle strength and lubrication of the joint capsule, which can lead to experiencing pain and pathological dysfunctionality [11, 12, 13]. Underestimation of OA symptoms is common, especially in young subjects. Joint pain can often be traced to poor posture, trauma, or aging. For this reason, treatments such as exercise and physical therapy are neglected rather than strictly adopted. Patients with OA are advised to join exercise programs, although involvement is often very low. Maintaining awareness of the severe consequences of inactivity and the benefit of exercise is essential, especially in the presence of a musculoskeletal disorder.

Human Activity Recognition (HAR) is a scientific approach aiming to collect data from various human activities such as walking, running, sitting, driving, and other daily activities [14]. Inertial measurement systems and wearables, such as smartphones or smartwatches, embedded with a 3-axial accelerometer and 3-axial gyroscope sensors are suitable for this use. The availability of digital devices with integrated sensors has sparked a growing interest in their use in health care systems and sports science [15, 16]. This approach strengthens the ability to recognize human activities in controlled and uncontrolled environments differently from biomechanics laboratories, which can only perform these measurements in controlled settings. Several wearable devices on the market are valid for monitoring human physical health [17]. The smartwatch market has grown exponentially in recent years. Sales of these devices were approximately 9 million in 2016, 12 million in 2017, reaching 22.6 million smartwatches sold in the United States during 2020 [18]. The high rise in sales of wearable devices reflects the interest in tracking everyday activities in consumers' lives [18]. The automatic recognition of PA practice and the monitoring of daily gestures through digital devices produce measurements that have been associated with the health status of several pathologies and have provided suggestions for their management [19]. Wearable devices can monitor PA in clinical practice and scientific research, especially for a prolonged period, revealing unpredictable changes in the investigated population [20].

This review aims at highlighting recent relevant literature about the use of digital devices for monitoring levels of PA in patients with OA, discuss the harness of real-world data deriving from digital devices in the context of digital epidemiology, and provide recommendations for researchers and clinicians approaching the use of wearables to collect health-data.

## 2 Methods

In this narrative review, we included articles from recent literature, providing a balanced and comprehensive overview of the most innovative discoveries on exercise monitoring for osteoarthritic patients and the current trends on management and statistical analysis of digital data. Subsequently, the selected articles were discussed in the following categories: “Wearables”, “Smartwatch applications”, “Digital epidemiology”. Key words for literature search included osteoarthritis, knee OA, hip OA, physical activity, exercise, digital devices, smartwatches, digital epidemiology, digital devices. The searches were limited to studies published in English that included human studies related to the presented categories. Study designs included narrative, systematic and meta-analyses reviews, original articles and randomized controlled trials (RCTs). We excluded protocols, abstracts without a full article, papers that replicated data from another article. We started the literature search in October 2020 to March 2021 on PubMed, Scopus, Web of Science and Google Scholar. 56 sources met the eligibility criteria, considered appropriate for the purpose of the review. These included 23 narrative, systematic and meta-analyses reviews, 2 RCTs and 23 original articles 8 other sources (1 website, 7 Society and Health Authorities guidelines). Since clinical applications of wearable devices for monitoring physical activity in patients with OA and digital epidemiology represent two quite unchartered fields of research, not a sufficient number of original articles was available to initiate a systematic review, therefore a narrative scoping review was preferred.

## 3 Wearables to analyze physical activity in osteoarthritic patients

Physical activity is defined as any movement produced by the muscles that expend energy, but it can include moving during leisure time or running at 15 km/h. The suitability and affordable cost of the wearables can improve health analysis in both daily activities and sports practice conditions thanks to the prolonged data collection. Patients with musculoskeletal diseases such as low back pain, osteoarthritis, and rheumatic inflammatory diseases are not well predisposed to the practice of PA [21], although it is considered indispensable to reduce pain and hypo-functionality [22].

Farr et al. [23] conducted one of the first studies about using wearables for a prolonged time in patients with OA. They attached an accelerometer through a belt to the right hip and measured the time spent in moderate, vigorous, and moderate-to-vigorous. Only 30% of the examined group (255 patients) achieved the CDC/ACSM recommendations [24, 25]. The PA average minutes were moderate 23.6 ± 17.2 min, vigorous 0.95 ± 3.5 min, and moderate-to-vigorous 24.54 ± 19.1 min. These results reflected a critical scenario among OA patients since a small percentage achieved a minimum of 30 min/day of moderate to vigorous PA.

A prospective study conducted by Morcos et al. [26] recruited 122 patients with hip OA scheduled for total hip arthroplasty, observed a positive correlation between PA levels and UCLA Activity score, Western Ontario and McMaster Universities Arthritis Index (WOMAC), Pain Catastrophizing Scale (PCS), Short-Form Health Survey (SF-12) and Harris Hip Scale (HHS). All patients wore a wristband activity tracker, Fitbit, for 24/7 consecutive days prior to their scheduled surgery. The results showed, moreover, that the mean number of steps per day was 5721 ± 3920. In line with the criteria by Tudor-Lock [27, 28], which classifies as sedentary those who accomplish less than 5000 steps per day, 51% of the participants would be considered sedentary. According to their results, measuring the PA levels can predict functional recovery after total hip arthroplasty, making wearables valuable tools for healthcare professionals.

The psychosocial aspects can benefit from digital supports because the patients feel more involved in the surgical/rehabilitation program. Long times and delays in functional recovery often arise from a lack of communication with the doctor or the fear of pain. These devices can improve trust, reduce recovery times and enhance the cooperation between doctor and patient, whereas they are monitored during daily life. In terms of adherence, the OA patients can accept to wear a device, allowing an objective assessment of PA during everyday activities.

## 4 Smartwatch applications to monitor osteoarthritic patients

Two research groups analyzed the feasibility and acceptability of smartwatches by utilizing two different applications (apps), KOALAP [29] and ROAMM [30, 31, 32], among knee OA patients. These two apps send, during the day, a survey to the consumer to evaluate the presence of pain, fatigue, falls, and activities practiced. Furthermore, the accelerometer counts daily steps as common smartwatches. These apps communicate with a specific online server, providing a reliable approach to remote personal health monitoring when worn.

Mardini et al. [31], analyzed the effectiveness of ROAMM (Real-time Online Activity and Mobility Monitor) data from 19 participants for 15 days classified in low and high OA pain groups. During the daily activities, the participants were surveyed in a random time window. The internet connection provided the data collection in real-time while the GPS recorded their location every 15 min to elaborate their travel pattern. The results showed a pain intensity range of 0–8 (highest reported value) and a valuable difference in GPS records between high and low pain groups. Pain intensity was significantly associated with the traveled area, reporting that each point of increase in mean pain intensity was associated with a decrease in the area walked by 3.06 km. The analysis of GPS data, along with pain intensity, can provide a suitable approach to understand the behavior of individuals and, therefore, suggest the best and personalized healthcare approach to use.

Beukenhorst et al. [29] employed the KOALAP (Knee OsteoArthritis: Linking Activity and Pain) app to study the daily activities of 26 participants for 90 days. The system was set up to trigger 4–5 questions about knee pain and quality of life. Unlike the previous study, these questions were administered within a specific time window, with a response time of 10 s per question, and the raw data were collected once the smartwatch was placed in charge. A baseline and follow-up questionnaire were administered about participants' experience with wearables and the relationship with knee OA. Participants wore the smartwatch 73% (81/90) of the days, for average daily usage of 11 h. The authors focused on psychosocial adherence to the program to discriminate the effectiveness of this approach. Patients found it interesting to learn more about the relationship between pain and activity. They showed high adherence to daily surveys, suggesting that pain questions could be collected more frequently to provide a detailed pain history. However, administering the survey within a specific time frame was reported to interfere with daily activities.

Psychological involvement may be crucial to increase the interest of patients in their health. Firstly, a patient may be afraid to walk because of pain onset. The smartwatch can measure the distance travelled to make the patient aware of the exact moment in which the pain occurs. Secondly, the patient could show higher adherence to the project when he/she feels constantly monitored, especially when there are no expectations. On the contrary, those with high expectations may become skeptical when the pain occurs in different circumstances aside from walking, e.g., sitting or standing.

## 5 The use of wearables to increase physical activity levels

The lack of knowledge of physical activity as healthy support can negatively influence the initiation and perpetuation of its practice. In this scenario, Davergne et al. [33] evaluated through a meta-analysis the efficacy of wearable devices to increase PA behavior in patients with rheumatic and musculoskeletal diseases. They included studies that used both common wearables (pedometer) and advanced wearables (smartwatch, fitness-tracker) for a short period, 0–8 weeks, or long period, > 8 weeks. Participants wearing the devices demonstrated greater adherence to training plans versus control groups; for example, they increased their daily steps for an average of 1,520 steps and achieved 16 min of moderate-to-vigorous PA. Those patients increased their fitness levels because they recognized the smartwatch as helpful in analyzing progress or any conditions that could lead to the onset of pain. Patients with OA are generally used to performing about 10 min of vigorous activity [34]; instead, these results suggest the efficacy of wearables to motivate patients to increase their PA levels.

Li et al. [35] enrolled 51 participants with knee OA and evaluated the results of a 12-week PA program through a smartwatch and accelerometer, Fitbit, and SenseWear Mini, respectively. During weeks 1–8, a personal trainer followed the participants and helped them with a phone call to change their PA where necessary. During weeks 9–12, participants had to continue their activity without a call from the personal trainer, although they could still email him to ask questions. Data present an increase of moderate-to-vigorous PA from baseline to week 13 of 10 min. Patients underwent successive follow-ups, attesting a constant increase of PA levels. In this scenario, wearables to monitor and personal trainer counseling resulted in effective support and motivated the patients to exercise and maintain a more active lifestyle.

## 6 Digital epidemiology

The spread of the Internet and the use of digital devices, e.g., smartphones and wearables, are rapidly introducing a new methodological approach for studying real-world phenomena. Clinical practice is experiencing an escalating transition from manual to automatized data collection. Therefore, as clinical information is progressively stored digitally, manipulating the data is easier and more accessible to other professionals. Collecting data from popular devices allows reaching an enormous number of people. It paves the way for the concept of "Digital Epidemiology" broadly and quickly defined as the epidemiology that uses digital data. Marcel Salathé, currently an associate professor at the École Polytechnique Fédérale de Lausanne (EPFL), Switzerland, suggests another well-thought definition: "Digital Epidemiology is epidemiology that uses data that was generated outside the public health system, i.e., with data that was not generated with the primary purpose of doing epidemiology" [36]. This definition cleverly focuses on a creative way to analyze existing data, whether normally generated through the daily use of digital devices (e.g., posts on social media, geotracking) or for other purposes (e.g., apps for exercise or consumption of calories, electronic medical and pharmaceutical records). It allows seeking and recognizing those types of data generated outside of public health that may be available and suitable for epidemiological studies, laying the foundation for "worldwide-based cohort studies".

**Figure 1:**
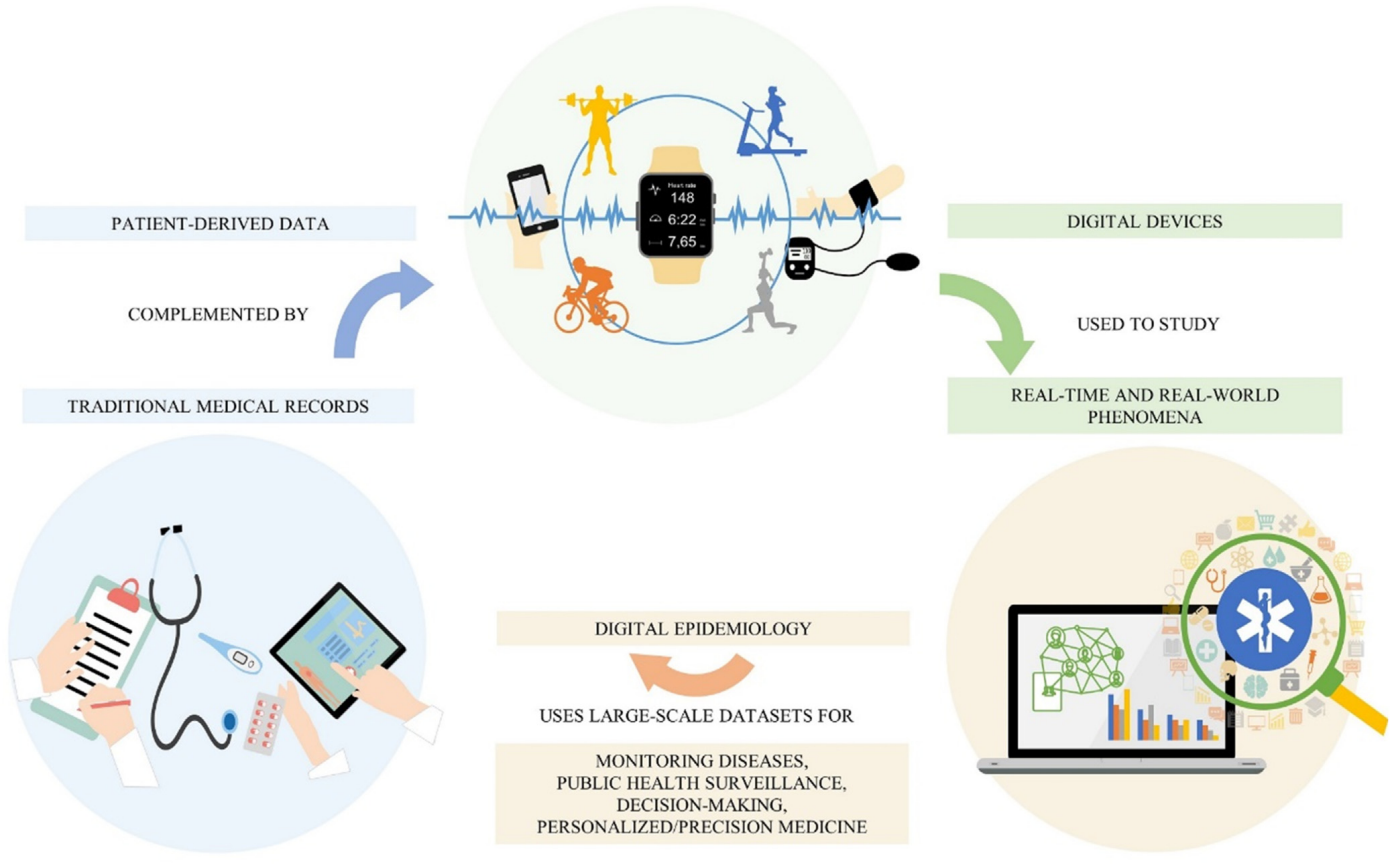
Overview of the relationship between traditional medical records, digital devices and digital epidemiology, and its impact on health system and patient care.

Traditional medical records and self-reported questionnaires regarding the health status of the patients can be corroborated by digital patient-derived data to provide a more comprehensive picture of the clinical case [37] (Figure 1). As wearable for PA in OA subjects, digital sources, practical to retrieve relevant information from the patient, could also be represented by internet activity (social media, forums) [38], credit card payments (pharmacy and grocery purchases), dedicated mobile apps (fitness, mental state, sleep monitoring). Lippi et al. [39] estimated a great increase, in the next future, of the amount of digital epidemiological research, in the form of PubMed articles, based on Google Trends (i.e., the frequency of word research) such as official cancer statistics [40]. Park et al. [41] reviewed 109 research articles that used digital data for epidemiological purposes by identifying health topic domains combined with different data sources. Health professionals can use digital supplementing data to elaborate a well-suited treatment to handle specific disease symptomatology, progression, or therapy outcomes. Instead, the scientific community can observe the influence of different behaviors or risk factors among large populations. In this context, sedentary behavior is one of the risk factors for OA onset, although it is challenging to estimate and quantify during someone's lifetime, exclusively through routine clinical visits and questionnaires. A retrospective investigation through digital data would be a helpful asset to the diagnostic process. Researchers can use sensors, accelerometers, and gyroscopes for longitudinal studies concerning the physical ability and PA of the patient during the day, especially in pathological conditions, such as OA, affecting the musculoskeletal system. Although this method of investigation is still in its infancy, it can spread over different medical areas such as neurodegenerative, psychiatric, or metabolic disorders [42, 43].

**Figure 2:**
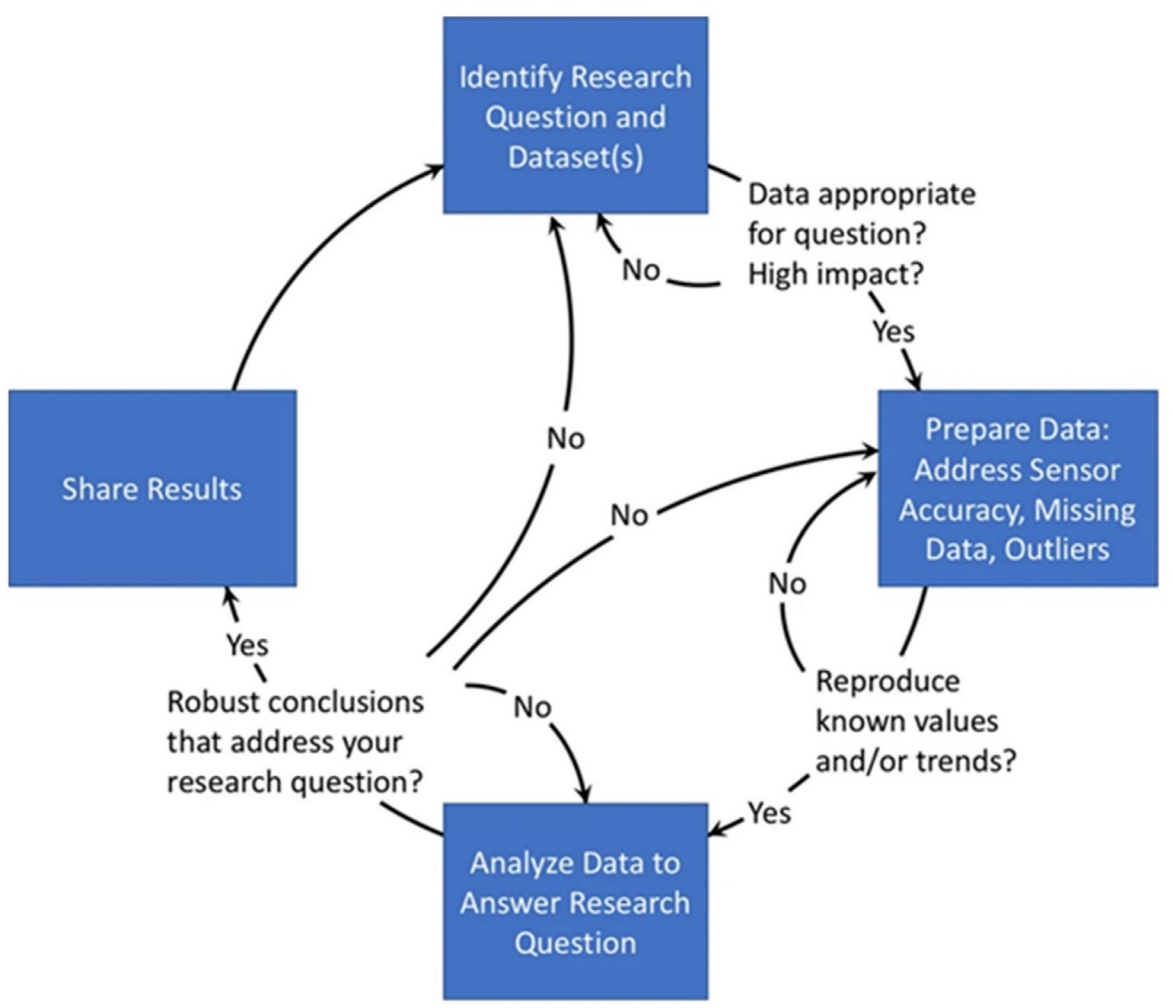
Workflow for analyzing large-scale datasets from commercial devices to provide epidemiological insights. Source: Hicks et al. [44]. Creative Commons Attribution 4.0 International License http://creativecommons.org/licenses/by/4.0/.

The intensity, duration, type, and frequency of PA, even simply walking, can draw attention to a wide range of health behavioral patterns, connected to mood changes or sleep disturbances. However, analyzing large-scale datasets requires data and medical science expertise to answer epidemiological questions about health issues. Hicks et al. [44] provided valuable guidelines for harnessing a large volume of data from smartphone apps and wearable devices relating to PA and other health behaviors and addressing the limitations concerning the analysis methods (Figure 2). The authors also outlined several common potential sources of error: complex intrinsic nature of the data because collected without a specific aim; missing data owing to measurement inaccuracy; different expectations about data-sharing partnerships between Academia and Industry.

## 7 Advantages and bias of metadata

As with any new type of information, patient-generated data and out-of-lab settings need further research to standardize the collection method. Large-scale studies, for example, have been conducted through the use of wearable devices by the mobile health company Azumio [45, 46]. They realized a low-cost app, e.g., Argus, designed for every smartphone and suitable for studies involving a large cohort of subjects, especially when difficult to provide wearable sensors [47, 48]. Mobile phone metadata have been already used effectively to monitor sleep [49], emotional states [50], transmission of diseases, such as malaria [51], and viruses [38], and to predict poverty and wealth in countries [52]. The obvious advantage of this device is its widespread use, counting smartphone owners in 69% of the population in developed countries and 46% in developing economies [46].

Although the ease with which these devices are commonly found in the population is an incentive to use them, their validity is not free from bias. Studies based on patient-derived digital data should describe the characteristics of the people examined to allow for good clustering of data and minimal variation between large samples. Selection bias can occur, as users may not represent a homogenous population, and information about gender, age, geographic location, socioeconomic status, race/ethnicity could lack; sensitivity or robustness testing is suggested [44]. Furthermore, wearable devices and smartphones need to be validated as digital tools, supporting the translation from the traditional tools used in common medical practice. A high rate of operator error and missing data should be considered as the patients themselves are responsible for the correct use of the devices. Long-term monitoring of patients is an attractive window of observation for physicians and researchers, even if these long times can lead patients to incorrect use of the device or withdrawal from the study. Positive feedback, self-management, and self-awareness can increase the program's adherence and reduce the withdrawal rate [53]. For example, PA monitor apps have a persuasive interface that reinforces and motivates attitudes to achieve or keep up with goals [54].

One drawback imposed by the use of digital devices is related to the mean age of the patients. Indeed, the elderly are discouraged from using applications and software; therefore, the subjects' age should also be considered, especially for OA disease which mostly involves the elders. However, this obstacle is most likely evident in the current historical period, when the older population struggles to adapt to the rapid technological advancements of modern electronics.

## 8 Data privacy

Finally, specific guidelines for consent, data processing, and international security standards are fundamental to maintaining public trust, strengthening data privacy, and providing secure access to personal data [55]. On the contrary, the current trend of major internet services to strictly protect their data ownership may slow down the spread and growth of digital epidemiology, limiting the open access for researchers and public health organizations [36].

In the context of the COVID-19 outbreak, M.M. Mello and C.J. Wang [56] raised ethical issues linked to digital epidemiology, sustaining a powerful concept: "these new uses of people's data can involve both personal and social harms, but so does failing to harness the enormous power of data to arrest epidemics".

## 9 Guidelines to monitor physical activity through wearables

**Figure 3:**
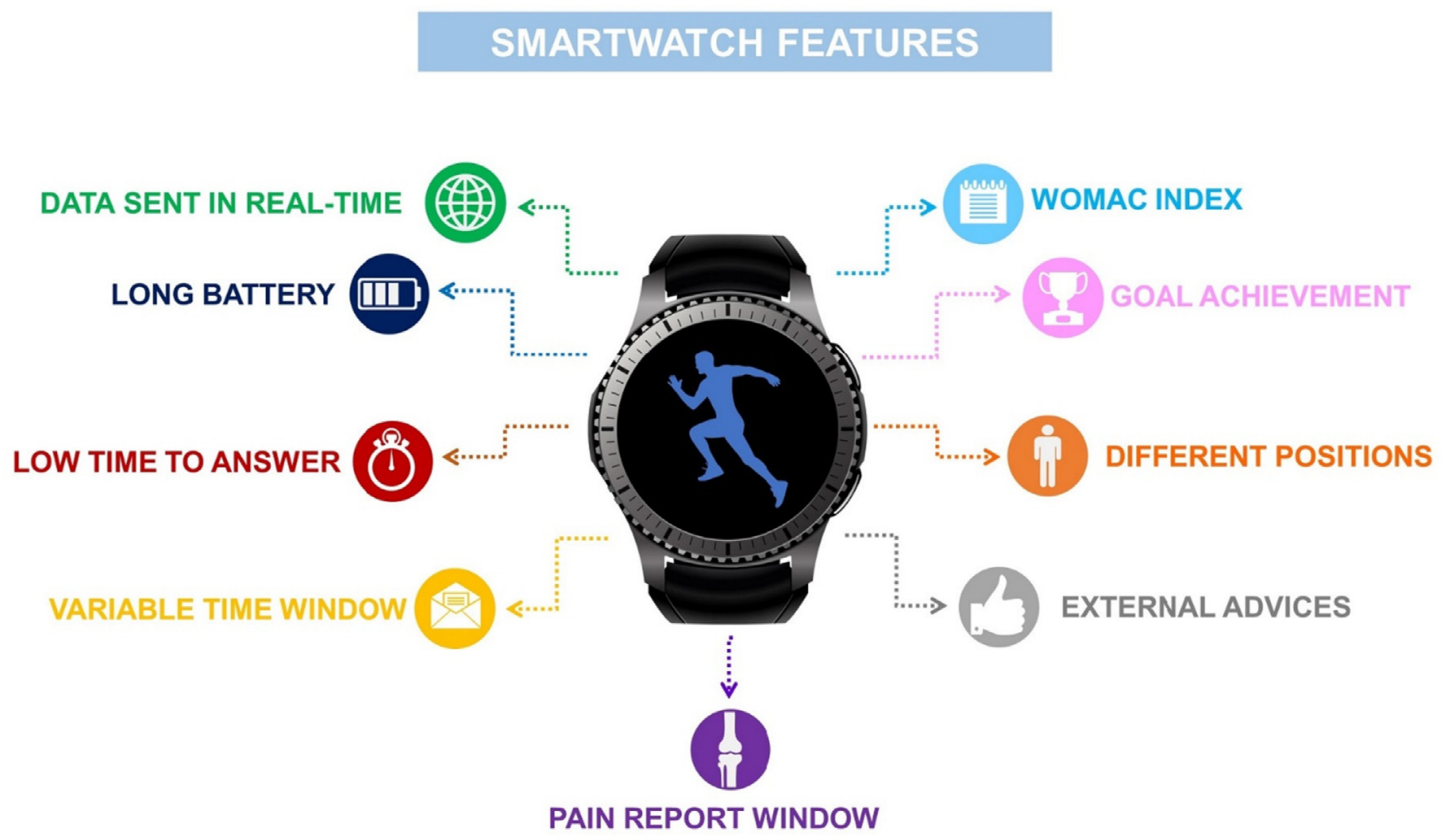
Recommended features to consider in the use of smartwatches for assessment of PA in OA patients.

Remote movement analysis can be as valuable as controversial, especially concerning patients who are not prone to technological advances. In the health promotion field, digital support can reach consumers in any way, via mobile phone or smartwatch. They represent a common, feasible, and low-cost tool for monitoring daily activities and PA for clinical and research purposes (Figure 3). In this review, we suggest the following guidelines when approaching the PA levels in OA analysis:

• Uploading data collected from smartwatches in real-time rather than during charging, preventing their loss.
• Preventing taking off the smartwatch or losing data through more extended battery life.
• Sending a maximum of 5 questions per day without a specific time frame, allowing the participants to answer at their convenience.
• Questions must be quick and easy to answer, taking up to 10 s.
• Patients can comment on their pain so that they can communicate their perceptions better.
• Analyzing different circumstances, e.g., sitting or standing, whereas these positions might cause severe pain.
• Introduce a simplified version of the WOMAC index to assess the OA physical functioning, administered through the smartwatch.

The following factors have to be considered to enhance the quality of physical activity:

• Increase motivation to perform PA by consulting personal achievements and progress.
• Strengthen adherence through the counseling of a personal trainer or physical therapist.
• Customize PA programs based on the subject's pain or difficulties.

## 10 Perspective

Daily habits, like performing PA or sleeping, have a renowned effect on musculoskeletal, neurological, cardiovascular systems, and overall wellness. However, until the advent of modern technology, these behaviors were difficult to observe and quantify during their lifetime. Collecting the data from smartphones and wearables is a valuable method to study real-time and real-world habits, especially in subjects with diseases. This approach can stimulate significant changes towards a healthier lifestyle in people suffering from painful conditions such as OA to reduce pharmacological and surgical interventions and slow the progression of the disease; with personal, social, and economic gains. More specifically, the studies reported in here highlight the advantages in using digital devices in OA patients to encourage them to maintain and promote exercise. Finally, digital epidemiology has the conditions to be considered a preliminary tool for observing phenomena related to the health sphere, such as outbreaks of the disease, therapeutic effects of the medications, health surveillance, or how OA is perceived by the general public earlier than conventional health epidemiology. A fruitful collaboration between biomedical researchers and data scientists will be needed to exploit the exponential volume of information, directing commercial apps and devices towards improving health at the individual, group, and population levels.

## Declarations

### Author contribution statement

All authors listed have significantly contributed to the development and the writing of this article.

### Funding statement

This work was supported by University Research Project Grant (PIACERI Found – NATURE-OA-2020-2022), Department of Biomedical and Biotechnological Sciences (BIOMETEC), 10.13039/501100004505University of Catania, Italy.

### Data availability statement

No data was used for the research described in the article.

### Declaration of interests statement

The authors declare no conflict of interest.

### Additional information

No additional information is available for this paper.
